# Utilizing medical thoracoscopy for the diagnosis of B‐cell lymphoma presenting with pleural effusion: A case series

**DOI:** 10.1002/rcr2.70061

**Published:** 2024-11-19

**Authors:** Nai‐Chien Huan, Khai Lip Ng, Larry Ellee Nyanti, Jing Yi Khaw, Jiun Hang Lee, Nur Husna Mohd Aminudin, Dahziela Yunus, Nusaibah Azman, Maryam Ahmad Sharifuddin, Hema Yamini Ramarmuty, Kunji Kannan Sivaraman Kannan

**Affiliations:** ^1^ Department of Respiratory Medicine Queen Elizabeth Hospital Kota Kinabalu Malaysia; ^2^ Department of Medicine Melaka Hospital Melaka Malaysia; ^3^ Medical Department, Faculty of Medicine and Health Sciences University Malaysia Sabah Kota Kinabalu Malaysia; ^4^ Department of Pathology Queen Elizabeth Hospital Kota Kinabalu Malaysia; ^5^ Department of Pathology Melaka Hospital Melaka Malaysia

**Keywords:** lymphoma, malignant effusion, medical thoracoscopy, pleural effusion

## Abstract

A third of patients with non‐Hodgkin's lymphoma (NHL) develop pleural effusion during the disease course for various reasons. In most cases, lymphoma‐related pleural effusion is a manifestation of widespread systemic disease, signifying a high tumour burden and therefore, a poorer prognosis. On the other hand, primary pleural lymphomas (PPLs) exhibit exclusive or dominant involvement of serous cavities, without detectable solid tumour masses. PPL is an uncommon disease and is of two types: primary effusion lymphoma (PEL) and diffuse large B‐cell lymphoma associated with chronic inflammation (DLBCL‐CI). PPLs not related to PELs and DLBCL‐CIs are exceedingly rare. Herein, we describe four patients with biopsy proven B‐cell NHL. One had no extra‐pleural involvement at the time of diagnosis, indicating PPL. In all cases, histopathological examination of pleural biopsies obtained via medical thoracoscopy (MT) were crucial in clinching the final diagnosis. Clinicians are alerted to the potential relationship between exudative effusion and NHL as well as the role of MT in the diagnosis of B‐cell NHL.

## Graphical abstract

In this case series, we describe four patients with medical thoracoscopy (MT) guided biopsy‐proven B‐cell NHL. All initially presented with unexplained pleural effusion. One patient had no extra‐pleural involvement at the time of diagnosis, indicating PPL. PPL is an uncommon disease and is of two types: primary effusion lymphoma (PEL) and diffuse large B‐cell lymphoma associated with chronic inflammation (DLBCL‐CI). PPLs not related to PELs and DLBCL‐CIs are exceedingly rare. Clinicians are alerted to the potential relationship between exudative effusion and NHL.

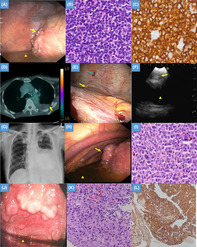

## INTRODUCTION

Pleural effusion is a common complication of non‐Hodgkin's lymphoma (NHL). 20%–30% of patients with NHL develop recurrent pleural effusion during the disease course,^1^ usually in the setting of systemic disease with a high tumour burden.^2^ Direct infiltration of the pleura and impaired lymphatic drainage are key mechanisms behind pleural effusions in Hodgkin's lymphoma and NHL, respectively.^3^ A sub‐group of lymphomas, termed primary pleural lymphomas (PPLs) exhibit exclusive or dominant involvement of serous cavities, without detectable solid tumour masses. PPL is a rare disease and is subdivided into two types, namely primary effusion lymphoma (PEL) and diffuse large B‐cell lymphoma associated with chronic inflammation (DLBCL‐CI).^4^ In this series, we describe four patients with medical thoracoscopy (MT) guided biopsy‐proven B‐cell NHL. All initially presented with unexplained pleural effusion (Table 1). One patient, case 2, had no extra‐pleural involvement at the time of diagnosis, indicating PPL. In all cases, histopathological examination of pleural biopsies obtained via MT were crucial in reaching the final diagnosis.

**TABLE 1:** Clinical characteristics and outcomes of patients with lymphoma‐related pleural effusion.

Case number	Case 1	Case 2	Case 3	Case 4
Age (years), Gender	71, male	80, female	72, male	62, male
Side & Size of effusion	Left, One‐third of hemithorax	Left, One‐third of hemithorax	Left, Half of hemithorax	Right, More than two‐thirds of hemithorax
Presence of extra‐pleural disease	Yes, posterior mediastinal mass with multiple enlarged abdominal lymph nodes	No	Yes, enlarged spleen with multiple enlarged abdominal lymph nodes	Yes, anterior mediastinal mass with partial compression of the right main bronchus
Pleural fluid: Colour LDH, U/L Protein, g/L ADA, U/L	Colour: yellowish serous	Colour: yellowish serous	Colour: yellowish serous	Colour: yellowish serous
	LDH: 198	LDH: 202	LDH: 212	LDH: 1975
	Protein: 48	Protein: 60	Protein: 52	Protein: 37
	ADA: 22.8	ADA: 4.15	ADA: 12.34	ADA: 174.53
MT findings	Localized area of nodules on the diaphragm	Localized area of thickened pleura with brownish specks at the costophrenic angle	Multiple nodules on the diaphragm and along the parietal pleura adjacent to the costophrenic angle	Multiple lobulated parietal pleural masses
Histopathology description	Dense tissue infiltrates of mainly lymphoid cells with a fibrotic background stroma	Fibroadipose tissue with crushed areas displaying infiltrates of lymphoid cells, arranged in sheets and small aggregates	Fibrofatty tissue infiltrated by malignant lymphoid cells in sheets	Fibro‐collagenous tissue diffusely infiltrated by atypical lymphoid cells
Immunophenotyping	Positive for CD20, PAX5 with a focal expression of CD43, CD23, IgM, IgD, BCL2 (weak). Negative for CD30, CD5, cyclinD1, LEF1, BCL6, CD10, MUM1. Proliferative Ki‐67 30%.	Positive for CD20, CD79a, with central germinal centre formations containing CD10+/BCL6+/BCL2‐ cells with brisk Ki67 proliferative cells and localized compact CD21+ FDC meshwork.	Positive for CD20, BCL6, MUM1 (>30%). Negative for CD3, CD10, PanCK. Ki67 80%.	Positive for CD20, CD79a, PAX5, CD23, BCL‐2, MUM1. Negative for CD3, CD5, CD10, CD15, CKAE1/AE3, AKL, cyclin D1, calretinin.
Final diagnosis	Marginal zone B‐cell lymphoma	Low‐grade B‐cell non‐Hodgkin's lymphoma	Diffuse large B‐cell lymphoma	Primary mediastinal large B‐cell lymphoma
Treatment given	Opted to observe rather than chemotherapy. Underwent talc pleurodesis 4 months after diagnosis to address recurring effusion	No chemotherapy was given. Underwent another drainage of effusion 5 months after the initial diagnosis	Received various chemotherapy regimens but surveillance CT showed disease relapse. Required interim drainage of effusion 4 months after initial diagnosis.	Chemotherapy R‐EPOCH regime. Talc pleurodesis was performed.
Outcome	Remained stable 6 months after diagnosis	Remained stable 9 months after diagnosis	Passed away due to neutropenic sepsis 1 year after initial diagnosis	No fluid recurrence 2 months after diagnosis

Abbreviations: ADA, adenosine deaminase; HIV, human immunodeficiency virus; LDH, lactate dehydrogenase; MT, medical thoracoscopy; R‐EPOCH, rituximab, etoposide, prednisolone, vincristine, cyclophosphamide, and doxorubicin.

## CASE SERIES

### Case 1

**FIGURE 1:**
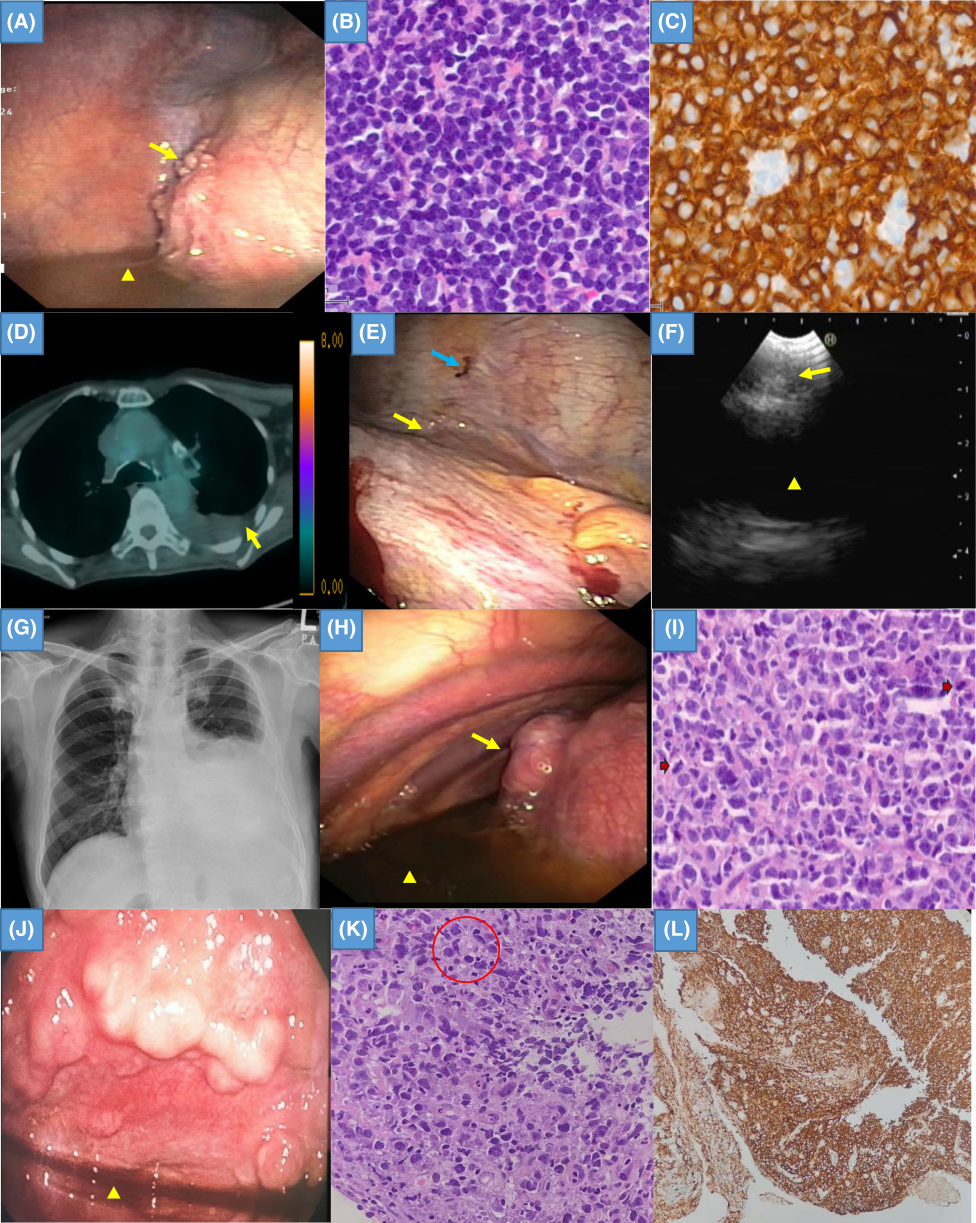
(A) Medical thoracoscopy (MT) image showed a localized area of nodules on the diaphragm (yellow arrow) and surrounding pleural effusion (yellow triangle). (B) Biopsy showed diffuse sheets of small to medium‐sized lymphoid cells (Haematoxylin and Eosin, H&E stain, ×400 magnification). (C) The lymphoid cells stained positive to CD20 (×400 magnification). (D) Fluorodeoxyglucose‐Positron Emission Tomography (FDG‐PET) scan showed effusion (yellow arrow) with mild activity in the mediastinal lymph nodes. No masses were detected in the thorax or elsewhere. (E) A focal area of nodular and thickened pleura (yellow arrow) with brownish spots (blue arrow) was noted at the costophrenic angle during MT. (F) Endobronchial ultrasound (EBUS) showed a 6 mm right lower paratracheal (station 4R) node with a central hilar structure (yellow arrow). The underlying superior vena cava was marked with a yellow triangle. The 4R node was sampled, showing lymphocytes without malignancy. (G) Chest radiograph showed moderate left pleural effusion occupying half of the hemithorax. (H) Multiple nodules were seen on the diaphragm (yellow arrow) and along the parietal pleura adjacent to the costophrenic angle during MT; pleural effusion was marked with a yellow triangle. (I) Haematoxylin and Eosin stain at ×40 magnification shows malignant moderate‐to‐large sized lymphoid cells arranged in sheets. Mitotic figures were pointed with red arrows. (J) MT revealed multiple lobulated parietal pleural masses with pleural effusion (yellow triangle). (K) Pleural biopsy showed that the atypical lymphocytes are intermediate to large in size, displaying pleomorphic nuclei, hyperchromasia, and occasionally prominent nucleoli. Mitotic figures (red circle) and cellular apoptosis were observed (H&E stain, ×400 magnification). (L) The lymphoid cells stained positive to CD20 (×100 magnification).

A 71‐year‐old gentleman with underlying diabetes mellitus and hypertension presented with cough, weight loss, anorexia, and worsening dyspnoea for 2 weeks. He never smoked and had no family history of malignancy. Physical examination and chest radiograph demonstrated left pleural effusion. A computed tomography (CT) scan revealed a posterior mediastinal mass with multiple abdominal lymphadenopathies. Thoracentesis revealed an exudative pleural effusion with pleural fluid lactate dehydrogenase (LDH) and protein of 198 U/L and 48 g/L respectively. His pleural fluid adenosine deaminase (ADA) was normal (22.8 U/L). Pleural fluid cultures and cytology were negative. MT showed a localized area of nodules on the diaphragm (Figure 1A). Biopsies revealed dense lymphocytic infiltration which stained positive for marginal zone B‐cell lymphoma. Immunohistochemistry confirmed the diagnosis with positivity for CD20 (Figure 1B,C). He underwent talc pleurodesis to address his recurring effusion 4 months after the initial diagnosis. He remained well with no fluid recurrence for 6 months and opted to monitor his lymphoma rather than pursue chemotherapy.

### Case 2

An 80‐year‐old woman with underlying hypertension, stage three chronic kidney disease, and depression presented with a dry cough and increasing shortness of breath for the past month. A chest radiograph revealed left pleural effusion, occupying about one‐third of the left thoracic cavity. CT thorax and whole‐body Fluorodeoxyglucose‐Positron Emission Tomography (FDG‐PET) demonstrated effusion with mild avidity in the mediastinal lymph nodes. No masses were detected in the thorax or elsewhere (Figure 1D). Her pleural fluid was exudative with pleural fluid LDH at 202 U/L and pleural fluid protein at 60 g/L. Her pleural fluid ADA was normal (4.15 U/L). A focal area of nodular and thickened pleura with brownish spots was noted at the costophrenic angle during MT (Figure 1E). Biopsy results indicated fibro‐adipose tissue with areas of disruption, revealing lymphoid cell infiltrates arranged in sheets and small clusters. Immunohistochemical analysis confirmed a low‐grade B‐cell non‐Hodgkin's lymphoma. She also underwent linear endobronchial ultrasound (EBUS) to examine her mediastinal nodes. Most nodes measured less than 5 mm, but her right lower paratracheal node measured 6 mm and was sampled, showing lymphocytes without malignancy (Figure 1F). Choosing to monitor her condition instead of receiving chemotherapy, she underwent drainage of her pleural effusion 5 months post‐diagnosis. The effusion has not recurred at the time of writing, 9 months after the initial presentation.

### Case 3

A 72‐year‐old gentleman with dyslipidaemia complained of dry cough, weight loss and night fever for 2 months. He never smoked and had no personal or family history of malignancy. Chest radiograph showed moderate left pleural effusion occupying half of the hemithorax (Figure 1G). CT demonstrated an enlarged spleen with multiple enlarged abdominal lymph nodes. Thoracentesis revealed an exudative pleural effusion with pleural fluid LDH and protein of 212 U/L and 52 g/L, respectively. His pleural fluid ADA was normal (12.34 U/L). Pleural fluid cultures and cytology were negative. Multiple nodules were seen on the diaphragm and along the parietal pleura adjacent to the costophrenic angle during MT (Figure 1H). Pleural biopsy results showed fibrofatty tissue infiltrated by malignant lymphoid cells in sheets. Immunohistochemical analysis was consistent with diffuse large B‐cell lymphoma (Figure 1I). The patient initially received CHOP (cyclophosphamide, doxorubicin, vincristine, and prednisolone) chemotherapy with rituximab, followed by multiple regimens including GCD (gemcitabine, carboplatin, and dexamethasone), GemOX (gemcitabine and oxaliplatin), ICE (ifosfamide, carboplatin, and etoposide) with obinutuzumab, and finally DHAP (dexamethasone, cytarabine, and cisplatin), as the disease proved refractory. Surveillance CT scans showed disease relapse, with progressive pleural involvement. Four months after his initial diagnosis, he required drainage of his pleural effusion. He succumbed to complications from neutropenic sepsis a year later.

### Case 4

A 62‐year‐old gentleman, ex‐smoker of 10 pack years with hypertension, dyslipidaemia and ischaemic heart disease presented with a two‐month history of weight loss and worsening dyspnoea. He had one previous hospitalization for a right pleural effusion but pleural fluid analyses were negative for infection and malignancy. Physical examination and chest radiograph showed recurrence of right pleural effusion. Diagnostic thoracentesis confirmed an exudative pleural effusion (fluid LDH and protein of 1975 U/L and 37 g/L, respectively). Pleural fluid cultures for tuberculosis, bacterial, and fungal organisms were negative. Pleural fluid cytology showed atypical lymphocytes suspicious of lymphoproliferative disorder. Pleural fluid ADA was raised (174.53 U/L). Due to limited access to urgent CT scans at our centre, we decided to proceed with MT, which revealed multiple lobulated parietal pleural masses (Figure 1J). Histopathological examination revealed fibro collagenous tissue diffusely infiltrated by medium to large‐sized atypical lymphoid cells, consistent with primary mediastinal large B‐cell lymphoma (Figure 1K,L). Post‐MT CT scan showed a large anterior mediastinal mass with partial compression of the right main bronchus and residual pleural effusion. Talc pleurodesis was performed to prevent effusion recurrence. Concurrently, he received chemotherapy (R‐EPOCH regime: rituximab, etoposide, prednisolone, vincristine, cyclophosphamide, and doxorubicin). He remained well with no fluid recurrence for 2 months and continued his treatment with the haematology team.

## DISCUSSION

Causes of lymphoma‐related pleural effusion include direct infiltration of the pleura by lymphoma cells, chylothorax secondary to lymphatic occlusion, radiation therapy, renal failure, cardiac failure, hypoalbuminemia, and pneumonia with parapneumonic effusion.^3^ T‐cell neoplasms, especially lymphoblastic lymphomas are more frequently linked to pleural effusions compared to B‐cell lymphomas.^5^ Within the B‐cell lymphomas, diffuse large B‐cell lymphoma carries the highest rate of pleural involvement at 60%, followed by follicular lymphoma at 20%.^6^ In our cohort, all cases of pleural effusion were associated with B‐cell lymphomas. The occurrence of pleural effusion in lymphoma patients often indicates a high disease burden and has been identified as a negative prognostic factor in various studies.^2^

Diagnosing lymphoma‐related pleural effusion can be challenging. The positive pleural fluid cytology rate for NHL varies significantly from 22.2% to 94.1% as lymphomatous cells can be sparse in the pleural fluid.^1^, ^3^ Besides, due to morphological similarities, distinguishing lymphomatous cells from normal or reactive lymphocytes in other tissues such as lymph nodes can be difficult in cytology.^7^ To improve diagnostic accuracy, ancillary techniques such as pleural fluid immunocytochemistry, flow cytometry, cytogenetics, and molecular genetics have been employed.^3^ However, these methods are often unavailable in resource‐limited settings. MT offers a promising alternative by allowing the collection of larger pleural samples with preserved tissue architecture. This approach can be useful when initial investigations such as pleural fluid cytology are inconclusive. Mehta et al. described a patient with adult lymphoblastic lymphoma diagnosed via MT when earlier investigations were negative.^8^ Similarly, Ng et al. reported two patients with MT guided‐biopsy‐proven myelomatous pleural effusion. Both patients had negative pleural fluid cytology results.^9^ Furthermore, MT is minimally invasive and can be performed under sedation, making it attractive for patients with multiple comorbidities. In our series, all patients had negative effusion cytological results and were ultimately diagnosed through histopathological examination of pleural biopsies obtained via MT.

PPL should be considered in cases with no signs of disease beyond the pleural space. PPL is a rare condition that falls into two categories: PEL and DLBCL‐CI.^4^ The exact pathogenesis remains unclear, but likely involves a complex interplay of viral infections and host immune responses leading to malignant transformation. PEL is linked to human herpesvirus‐8 (HHV‐8) and typically occurs in immunocompromised patients secondary to human immune‐deficiency virus (HIV) infection. On the other hand, DLBCL‐CI is associated with Epstein–Barr virus (EBV) and often presents as pleural lymphoma in patients with a history of pyothorax from old tuberculous pleuritis.^10^ Both PEL and DLBCL‐CI have poor prognoses, with median survival rates of 18 months^11^ and less than 12 months,^4^ respectively. Isolated B‐cell‐related PPL outside the contexts of PEL and DLBCL‐CI is rare and tends to follow a distinct clinical trajectory. A previous case series of eight patients with marginal zone lymphoma‐related PPL demonstrated an indolent disease course, in contrast to PEL and DLBCL‐CI.^12^ Notably, half of these patients achieved complete remission following local pleural treatments, such as pleurodesis, with or without systemic chemotherapy. Similarly, Zhang et al. documented a case of indolent B‐cell lymphoma‐related PPL where massive pleural effusion was the initial manifestation, and the patient remained stable on follow‐up without chemotherapy, similar to our second case.^13^ Other studies have reported that several untreated patients followed for 6 to 40 months showed no significant disease progression.^14^, ^15^

Elevated levels of ADA in pleural fluid, typically exceeding 30 U/L, are commonly used as an adjunctive test for diagnosing tuberculous pleural effusion (TBE) in endemic regions.^16^ However, high ADA levels can also be observed in lymphoma and myeloma‐related pleural effusions. Specifically, among effusions linked to lymphoma, elevated ADA levels are more frequently associated with T‐cell neoplasms than with B‐cell neoplasms.^17^ This difference is likely due to the higher ADA activity in T‐lymphocytes compared to B‐lymphocytes, which may explain why elevated ADA levels were not observed in all our patients. Despite variations, elevated ADA levels should not dissuade healthcare providers from utilizing other standard diagnostic procedures such as MT, particularly when clinical evidence does not align with TBE. In our fourth case, we proceeded with MT despite an elevated ADA level, as the patient lacked significant risk factors for tuberculosis.

In managing pleural effusion related to B‐cell lymphoma, treatment typically involves systemic chemotherapy, localized radiotherapy, and/or stem cell transplantation. Intrapleural rituximab^18^ has been described, but it is not yet widely adopted. Additionally, fluid management strategies like talc pleurodesis may be necessary for recurrent effusions that persist despite specific lymphoma therapies.

In summary, we reported four patients with NHL‐related pleural effusions diagnosed by MT. Although uncommon, clinicians should be aware of the relationship between unexplained pleural effusions and NHL. MT appears to be a safe, feasible, and minimally invasive tool for the diagnosis of lymphoma‐related pleural effusions when other initial investigations are non‐conclusive.

## AUTHOR CONTRIBUTIONS

Nai‐Chien Huan and Khai Lip Ng contributed to the design and implementation of the case report. Nai‐Chien Huan, Khai Lip Ng, Larry Ellee Nyanti, Jing Yi Khaw, and Jiun Hang Lee wrote the manuscript. Nai‐Chien Huan, Khai Lip Ng, Larry Ellee Nyanti, Nur Husna Mohd Aminudin, Dahziela Yunus, Nusaibah Azman, Maryam Ahmad Sharifuddin, Hema Yamini Ramarmuty carried out the procedures mentioned. Kunji Kannan Sivaraman Kannan supervised the project. All authors discussed and contributed to the final manuscript.

## CONFLICT OF INTEREST STATEMENT

None declared.

## ETHICS STATEMENT

The authors declare that appropriate written informed consent was obtained for the publication of this manuscript and accompanying images.

## Data Availability

The data that support the findings of this study are available from the corresponding author upon reasonable request.
